# Successful low-dosage thrombolysis of massive pulmonary embolism in primigravida: A case report

**DOI:** 10.1097/MD.0000000000012985

**Published:** 2018-10-26

**Authors:** Jiyang Liao, Fang Lai, Dongping Xie, Yun Han, Shutao Mai, Yanna Weng, Yan Zhang, Jiongdong Du, Gengbiao Zhou

**Affiliations:** a Postgraduate Student of the Second Clinical College of Guangzhou University of Chinese Medicine; b The Second Affiliated Hospital of Guangzhou University of Chinese Medicine; c Guangdong Provincial Hospital of Chinese Medicine; d Doctoral Student of the Second Clinical College of Guangzhou University of Chinese Medicine, Guangzhou, Guangdong, China.

**Keywords:** low-dosage thrombolysis, nonradiometric examinations, primigravida

## Abstract

**Rational:**

Thrombolysis in primigravida with hemodynamic instability is controversial, especially treatment with low-dosage recombinant tissue plasminogen activator (rtPA), and related studies are extremely rare. Here, we report the case of a 26-year-old primigravida diagnosed with an acute massive pulmonary embolism (PE) that prompted initiation of thrombolysis with low-dose alteplase.

**Patient concerns:**

The patient was admitted to the Emergency Department with chief complaints of a sudden onset of extremely dyspnea, chest tightness, and confusion over a 6-hour period. She was found to have significant dilation of her right ventricle, moderate pulmonary arterial hypotension, as shown by transthoracic echocardiography, and a typical S1-Q3-T3 pattern, as shown by electrocardiogram (ECG).

**Diagnosis:**

Acute massive PE in primigravida.

**Intervention:**

The patient underwent intravenous thrombolysis with a half dose of alteplase.

**Outcomes:**

The fetus lived through this severe event during the mother's stay in the Intensive Care Unit; however, surgical abortion was unexpectedly proposed due to long-term hypoxia and high-risk of relapse and exacerbation and was performed successfully after the agreement of her kin. The patient recovered gradually, and results of her laboratory tests and postsurgical, repeated contrast-enhanced computed tomography had normalized by her 3-month follow-up.

**Lessons:**

Administration of low-dosage alteplase in primigravida with hemodynamic instability is extremely rare and controversial; however, our case suggests that this treatment strategy is relatively safe and feasible. In addition, nonradiometric examination played a major role in the diagnosis of PE in this patient. Because radiation use is contraindicated during pregnancy, these examinations could be the first choice for pregnant patients with suspected PE.

## 1 Introduction

Maternal death related to pregnancy is most often attributable to pulmonary embolism (PE), in developed countries.^[1]^ Venous thromboembolism (VTE) is thought to be “provoked” by interaction between patient-related and setting-related risk factors. Moreover, the rate of pregnant patients suffering from VTE is 5 times higher than that in nonpregnant women of similar age with respect to PE morbidity.^[2]^ However, the implementation of randomized controlled trials remains difficult in order to establish a consistent treatment program for use of thrombolysis during pregnancy and to verify the efficacy and safety of this unique treatment regimen. The European Society of Cardiology has recommended the initiation of thrombolysis when high-risk patients present with hemodynamic instability, in its 2014 acute pulmonary embolism treatment guidelines.^[3]^ Although there is growing evidence that heparin is a safe anticoagulant to administer during pregnancy,^[4,5]^ there have been no conclusive guidelines for the use of thrombolytic agents during the hemodynamic instability period of pregnancy. Adaptations with respect to the monitoring of thrombolytic agents may, therefore, require further investigation. Herein, we report a successful case of treatment with low-dose thrombolysis in high-risk APE in primigravida that occurred in the first trimester. In contrast to previous case reports, not only the patient was diagnosed with acute massive PE, which was confirmed by a protein S deficiency, but also, she was successfully treated using the rare treatment strategy of administering a low-dose thrombolytic agent.

## 2 Case report

**Figure 1:**
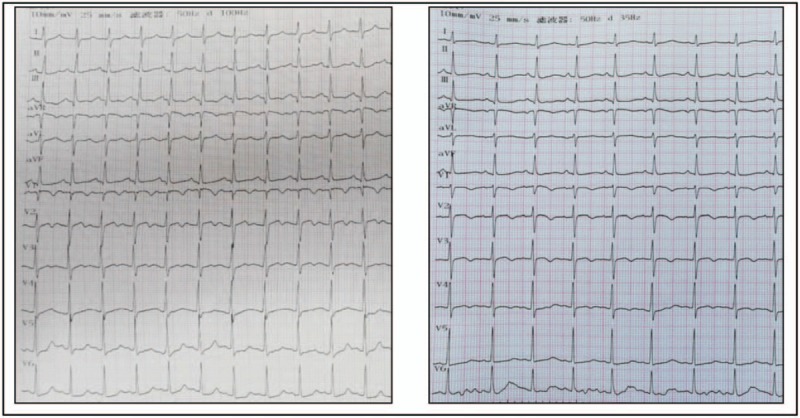
Electrocardiogram (ECG) on admission, before thrombolysis and 2 h after thrombolysis. ECG displayed the development of an S wave in Lead I, a Q and inverted T waves in Lead III in thoracic leads before thrombolysis on the left figures; ECG on the right figure returned to normal after thrombolysis.

A 26-year-old primigravida at 10 week's gestation was admitted to our emergency department with complaints of a sudden onset of extreme dyspnea, chest tightness, and confusion over a 6-hour period. No significant medical history or drug consumption was noted. She had dysphoria accompanied by tachycardia (141 beats/min) and tachypnea (42 breaths/min). Consistent with the peripheral blood oxygen saturation value, arterial gas analysis showed decompensated metabolic acidosis (pH: 7.216, PO_2_: 47.2 mm Hg, PCO_2_: 37.7 mm Hg, lactate: 6.10 mmol/L, and base deficit: −11.6 mmol/L) (Table 1). Endotracheal tube intubation and mechanical ventilation were initiated immediately. An electrocardiogram (ECG) was taken considering that her symptoms revealed an S1-Q3-T3 pattern particularly seen in PE (Fig. 1).

**Table 1:**
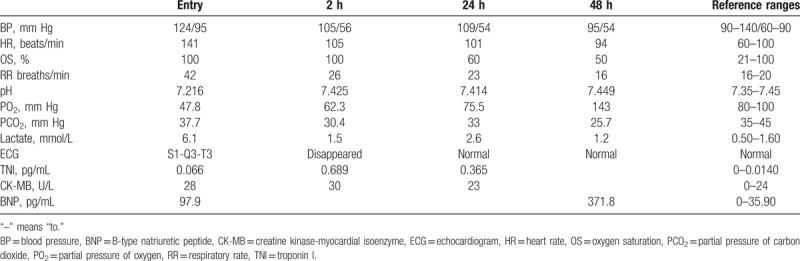
Clinical and laboratory test in the first 48 h.

**Figure 2:**
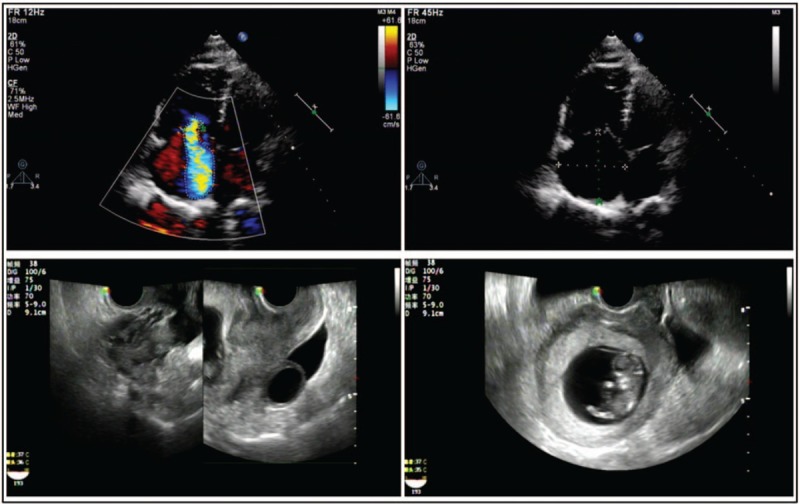
Ultrasonography on admission. Moderate-to-severe tricuspid regurgitation, and a distended right ventricle and atrium were revealed by the transthoracic echocardiography before thrombolysis. The fetal Doppler instrument revealed fetal heart 2 h after thrombolysis.

The patient was then transferred to the intensive care unit after central vein catheterization. Laboratory tests, including prothrombin time, activated partial thromboplastin time, international normalized ratio, fibrin degradation products, D-dimer, troponin I, serum electrolytes, and arterial blood gas, were obtained every 6 hours within the first 24 hours. The fetus had been at high risk of death before the mother was admitted to our hospital, due to the duration of acute anoxia. Informed consent for procedures that might cause fetal harm and worse, may require the necessity of an abortion was obtained when the kin reached an agreement with respect to the patient's condition. Transthoracic echocardiography revealed moderate-to-severe tricuspid regurgitation and a distended right ventricle. The right ventricle free-wall was hypokinetic, which was simultaneously accompanied by moderate pulmonary hypotension. The left ventricle was normal in size and function (Fig. 2). Because of the contraindication to the use of radiation, contrast-enhanced spiral computed tomography performance was delayed, as was catheter embolectomy. At this point, in view of the patient's life-threatening condition and to avoid the risk of metrorrhagia, thrombolytic therapy with a low-dosage of alteplase (10 mg loading dose and 40 mg pumped intravenously over 2 hours) was administered once informed consent was obtained. Sedation was continuously monitored. Bicarbonate was provided to correct metabolic acidosis, and administration of low-molecular-weight heparin (LMWH, enoxaparin, 6000 IU twice daily) commenced subsequently according to the prothrombin time for the first 48 hours. Following thrombolysis, reduction in heart and respiratory rates, as well as improvement in blood pressure and oxygen saturation in arterial gas analysis were observed. An ECG was repeated and showed reversal of the S1-Q3-T3 pattern after thrombolysis (Fig. 1). However, a serious reduction in fibrinogen (less than 0.5 g/L) was seen. Lyophilized human fibrinogen was infused to correct this treatment side effect, and the fibrinogen level returned to within the normal range within the first 24 hours. On the day following thrombolysis, we observed a heart rate of 105 beats/min, 96% oxygen saturation (invasive ventilation, 100% oxygen), and blood pressure of 105/56 mm Hg; however, a repeated arterial gas analysis revealed no significant change in lung perfusion, and hypoxemia remained (pH: 7.428, PO_2_: 64.3 mm Hg, PCO_2_: 29.9 mm Hg, lactate: 1.2 mmol/L, and base deficit: −3.5 mmol/L). Unfortunately, urinalysis revealed that severe hematuria was present, as sediment in the urine contained 508 red blood cells, as seen by the high-power-field of a microscope. However, the performance of a pelvic Doppler ultrasound showed a regular fetal heartbeat, normal placenta, and normal liquid presence (Fig. 2). At this point, treatment progress reached a stalemate among clinicians.

Maintenance of the conservative anticoagulation regimen was elected following multidisciplinary discussions. Clinicians from the Department of Cardiac and Thoracic Surgery suggested that the thrombus could be removed through surgical embolectomy or percutaneous catheter-directed treatment; however, the major limitation of this option was her family's wish, at this point in time, to prevent miscarriage, taking into account that it would be difficult for the fetus to tolerate the greater impact from surgery. Although predicting the recanalization of the lung perfusion remained problematic, the clinicians from Gynecology suggested that maintaining the conservative anticoagulation therapy might be the best option. Not only had the urinalysis shown severe hematuria, but also the patient's vital signs revealed a recovery potential. After comprehensive evaluation and careful consideration, we adopted the suggestion from Gynecology. We then advised the patient's family of the effects of long-term severe anoxia on the fetus, the high-risk of relapses, and exacerbation of PE; in view of that information, her family wished for us to maximize our efforts to save the mother's life and to avoid concomitant mental retardation of the fetus. Therefore, an induced abortion was unexpectedly proposed and was subsequently performed successfully. The natural history of PE markedly altered after the first 24 hours postoperatively; 2 arterial blood gas analyses showed that oxygen partial pressure had first risen to 78 mm Hg (invasive ventilation, 60% oxygen), and then to 143 mm Hg (invasive ventilation, 50% oxygen) (Table 1). A sequential high-flow nasal catheter was inserted successfully when invasive ventilation was weaned on the morning of the 2nd postoperative day.

No major bleeding was observed, and all laboratory test levels had gradually recovered to within normal limits, except for disturbing results of a protein S test—serum protein S activity had dropped to less than 16%. Given her severe condition, we considered that the deficit of this anticoagulant factor may have been involved in her unprovoked PE, while all her ultrasound results were negative for problems in her extremities and abdomen.

**Figure 3:**
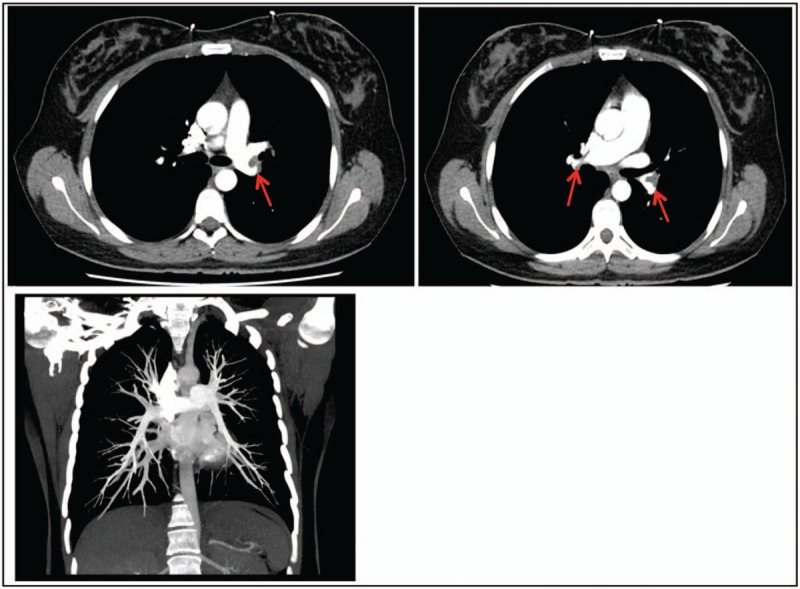
Contrast-enhanced computed tomography of the chest after thrombolysis, *red arrows* in the first column showed thrombi occupying the pulmonary arteries bilaterally before discharge, and the recanalization of the lung perfusion in the second column was observed after 3 months follow-up.

LMWH was subsequently changed to warfarin and new oral anticoagulants during the postoperative period. Complete disappearance of hypoxia and normalization of laboratory test results were observed in the following days, and she was subsequently discharged home in good condition 13 days after admission. Repeat postsurgery contrast-enhanced CT scan results were also consistent with the significant serum results, indicating that the thrombi had been substantially diminished in infarct size, when the recanalization of lung perfusion was evaluated at the 3rd month follow-up (Fig. 3). The study protocol approved by the hospital's Ethics Review Committee and complied with the tenets of the Declaration of Helsinki.

## 3 Discussion

A weight-adjusted dose of LMWH has been recommended for use in pregnancy;^[3]^ not only does the increasing experience with administering this drug testify to and endorse its safety, but also, the fact that it does not cross the placenta has been confirmed. Similarly, it has also been reported that thrombolytic agents are unlikely to cross the placenta, because their molecular weights are greater than 1000 Daltons.^[6,7]^ In 2014, the ESC suggested that thrombolytic agents only be used during pregnancy under high-risk conditions. However, randomizing pregnancy with thrombolytic therapy remains ethically unfeasible, not only because the data are limited, but also because therapeutic determinations vary based on the exact condition for which thrombolytic agent use is indicated. Moreover, the limited suggestions provided by the PE guidelines have primarily been made based on pooled and analyzed data obtained from case reports; therefore, clinicians should exercise caution when drawing conclusions in actual clinical situations. Gomes et al ^[8]^ studied 65 articles describing the use of thrombolytic agents during pregnancy and maternity and reported that the complication rate of thrombolytic agent use in pregnant women appeared to be the same as that in nonpregnant women. Twenty-five women suffering with PE during the gestational period were included in this review. Moreover, no randomized controlled trials were performed; only 4 patients received low-dose recombinant tissue plasminogen activator (rtPA ≤50 mg), and 1 presented with a minor hemorrhage at the puncture site. The incidence rate of complications following rtPA treatment appeared to be lower than that following the use of other thrombolytic agents. However, despite the statistical methods used to analyze the data, verifying results were not entirely possible, because clinical evidence from systematic research was absent.

Initiation of standard thrombolysis is recommended for use in patients with shock or hypotension. In our case, once the decision to administer thrombolytic therapy was made by agreement between our patient's kin and clinicians, the dosage was halved after considering the risks for metrorrhagia and other major bleeding. Her vital signs improved after the thrombolysis, and marked systematic improvement was seen after the first 24 hours following the abortion. However, considering the low level of protein-S activity, which decidedly induced the thrombus, and her own hypercoagulability, the marked decrease in fibrin catabolism could have led to the clinicians’ strong suspicion of thrombophilia. In addition, there existed concerns about placental abruption during thrombolytic therapy; however, this complication did not occur, while only a minor case of hematuria, which was too mild to result in a reduction of hemoglobin, was seen. A contraindication to repeated thrombolysis was determined due to the results of urinalysis and following a multidisciplinary discussion. For acute massive PE during pregnancy, surgical embolectomy, and percutaneous catheter-directed treatment should be considered carefully after weighing the advantages and disadvantages for both the fetus and the mother. Before performance of an induced abortion is proposed, maintaining the anticoagulation might be the best option. Meneveau et al ^[9]^ conducted a prospective single-center study to investigate the benefit of surgical embolectomy and repeated thrombolysis in patients who did not respond to initial thrombolysis. They studied 488 PE patients over a period of 10 years, and the results showed that mortality in the medical group was higher than that in the surgical group (10 deaths vs 1 death, respectively; *P* = .07), and any bleeding events after repeated thrombolysis resulted in fatalities. Therefore, the clinicians who had opted for surgery had obtained the best results in this study.

The management of massive PE with effective noninvasive therapeutic thrombolysis and the diagnostic methods used in pregnancy can vary dramatically. An ideal thrombolytic agent should induce local thrombus dissolution without systematic complications, and, as often as possible, use of ionizing radiation in making a diagnosis should be avoided when PE is suspected during pregnancy. In our case, the urgent circumstances required a quick diagnosis and timely treatment. Results of echocardiography, electrocardiogram, bedside ultrasonography, and laboratory testing were all consistent with the diagnosis of thrombolysis, therefore, prompting us to initiate thrombolysis without hesitation.

For the above-mentioned reasons, thrombolysis remains an essential and specific therapy to treat pregnant patients with hemodynamic instability. Moreover, in an effort to avoid unnecessary complications, such as minor or major bleeding in the genital tract or urethra, a dosage reduction in thrombolytic agents may be a safer option than standard dosages; however, this hypothesis requires further investigation, and additional studies must be conducted to challenge therapeutic and ethical problems.

## 4 Conclusion

Thrombolysis can be recommended as the optimal therapy to treat pregnant patients with PE and hemodynamic instability. However, thrombolytic treatment may be limited by, for example, ethical considerations and opinions from family members; therefore, further studies evaluating thrombolytic treatment remain difficult to conduct during pregnancy. As this case depicted, fibrinolytic activity was enhanced effectively with a half dose of rtPA and subsequent subcutaneous injection of LMWH. In contrast to similar studies, no major complications were observed in either the mother or fetus, and no major bleeding occurred. Therefore, this treatment regimen should be considered in similar cases to improve prognoses of both the mother and fetus.

## Acknowledgment

The authors thank their patient for providing consent to publish this case report.

## Author contributions

All authors participated in the clinical care of patient.

**Conceptualization:** Jiyang Liao, Yun Han, Yanna Weng, Fang Lai, Shutao Mai.

**Data curation:** Jiyang Liao, Jiongdong Du.

**Formal analysis:** Jiyang Liao, Yun Han, Dongping Xie, Fang Lai.

**Funding acquisition:** Fang Lai.

**Investigation:** Jiyang Liao.

**Methodology:** Jiyang Liao, Yun Han, Dongping Xie, Fang Lai, Yan Zhang, Gengbiao Zhou.

**Project administration:** Jiyang Liao, Dongping Xie, Fang Lai.

**Resources:** Jiyang Liao.

**Software:** Jiyang Liao.

Jiyang Liao orcid: 0000-0002-1189-2222.
